# COVID-19 experience of the major pandemic response center in the capital: results of the pandemic’s first month in Turkey

**DOI:** 10.3906/sag-2006-164

**Published:** 2020-12-17

**Authors:** Rahmet GÜNER, İmran HASANOĞLU, Bircan KAYAASLAN, Adalet AYPAK, Ayşe KAYA KALEM, Fatma ESER, Burcu ÖZDEMİR, Elif Mükime SARICAOĞLU, Müge AYHAN, Yeşim AYBAR BİLİR, Işıl ÖZKOÇAK TURAN, Deniz ERDEM, Nevzat Mehmet MUTLU, Turan BUZGAN, Bedia DİNÇ, Esragül AKINCI

**Affiliations:** 1 Department of Infectious Diseases and Clinical Microbiology, Yıldırım Beyazıt University School of Medicine,Ankara City Hospital, Ankara Turkey; 2 Department of Infectious Diseases and Clinical Microbiology, Ankara City Hospital, Ankara Turkey; 3 Department of Intensive Care, Ankara City Hospital, Ankara Turkey; 4 Department of Microbiology, Ankara City Hospital, Ankara Turkey; 5 Department of Infectious Diseases and Clinical Microbiology, University of Health Sciences, Ankara City Hospital, Ankara Turkey

**Keywords:** COVID-19, Turkey, epidemiology, prognosis, IL-6, mortality

## Abstract

**Background/aim:**

The aim of this study is to evaluate the epidemiological and clinical characteristics and parameters that determined the clinical course and prognosis of the COVID-19 patients admitted to Ankara City Hospital during the first month of the pandemic in Turkey.

**Materials and methods:**

SARS-CoV-2 PCR positive patients who were hospitalized between March 10 and April 10, 2020 were included.

**Results:**

Among 222 patients, mean age was higher in severe acute respiratory illness (SARI)/critical disease group (P < 0.001). Median time from illness onset to admission and presence of comorbidity, especially coronary artery disease and chronic obstructive pulmonary disease, were significantly higher in the SARI/critical disease group (P < 0.05). Cough and fever were the most common symptoms, while anosmia and loss of taste were observed in 8.6% and 7.7% patients, respectively. The mortality rate was 5.4%. A high neutrophil/lymphocyte ratio; low lymphocyte, monocyte, and platelet count; elevated liver enzymes; low GFR; and high levels of muscle enzymes, ferritin, and IL-6 on admission were found to be associated with SARI/critical disease (P < 0.05). Bilateral ground-glass opacity and patchy infiltration were more frequently seen in the SARI/critical disease group (P < 0.001). Patients older than 65 years had an 8-fold increased risk for development of SARI/critical disease.

**Conclusion:**

This cohort study regarding COVID-19 cases in Turkey reveals that older age, presence of comorbidity, bilateral infiltration on CT, high neutrophil/lymphocyte ratio, low monocyte and platelet count, elevated liver enzymes, low GFR, high levels of muscle enzymes, and high levels of ferritin and IL-6 on admission are predictors of SARI and severe disease.

## 1. Introduction

On December 31, 2019, the World Health Organization (WHO) China Country Office reported pneumonia cases of unknown etiology in Wuhan, Hubei Province, China. On January 7, 2020, the causative agent was identified as a new Coronavirus (2019-nCoV), which had not previously been detected in humans [1]. Later, the name of 2019-nCoV disease was accepted as COVID-19, and the virus was named SARS-CoV-2 because of its close resemblance to SARS CoV. After this date, the number of patients increased rapidly, and the WHO declared an “International Public Health Emergency” regarding the coronavirus outbreak at its meeting on January 30, 2020.

Although the world was caught unprepared to the sudden emergence and rapid spread of the COVID-19 outbreak, Turkey managed to postpone the emergence of the disease within its borders through the implementation of effective preventive measures until March 11, 2020, when the first case was detected. After cases seen in China, Italy, and Spain starting from January, Turkey executed a meticulous monitoring and evaluation process to decide, implement, and follow up with comprehensive and timely measures. These measures have given time to be prepared for both the community and the healthcare system in this pandemic. Furthermore, since the beginning of the pandemic, Turkish citizens infected with COVID-19 have been treated free of charge and all equipment, tests, kits, drugs that are used during diagnosis and treatment are provided by the Ministry of Health.

The aim of this study is to evaluate the epidemiological and clinical characteristics of the COVID-19 patients admitted to the Ankara City Hospital and to reveal the factors and parameters that determine the clinical course and prognosis of the patients. Ankara City Hospital, the largest hospital in Europe with a total of 3811 beds (723 of which are intensive care unit beds), employs 16,000 healthcare workers and is the major pandemic response center in Ankara.

## 2. Materials and methods

Patients older than 18 years who were hospitalized in Ankara City Hospital between March 10 and April 10, 2020, and who were found PCR positive for SARS-CoV-2 in oropharyngeal/nasopharyngeal swab samples or deep tracheal aspiration were included. Patients who had no follow-up for at least 14 days were excluded from the study.

Demographic features such as age, sex, place of residence, symptoms and onset time, travel history, comorbidities, physical examination, fever, and vital signs for clinical responses were recorded from the follow-up charts. Laboratory tests such as complete blood count, blood chemical analysis (including renal and liver function), coagulation parameters, acute phase reactants (ferritin, C-reactive protein (CRP), procalcitonin (PCT), and interleukin-6 (IL6)), and cardiac markers (creatin kinase (CK), CK-MB, myoglobin, cardiac troponins, and pro-BNP) in patients deemed necessary were also obtained. Both X-ray and computed tomography (CT) of the chest were used for radiological assessment. Control radiography and CT were performed in patients with clinical deterioration or in case of no response to the medical treatment.

We classified COVID-19 patients into five groups according to WHO guidelines (mild illness, pneumonia, severe pneumonia, acute respiratory distress syndrome (ARDS), and sepsis/septic shock). For comparisons, patients were divided into two categories (mild disease/pneumonia and severe acute respiratory illness (SARI)/critical disease). The critical disease category consisted of severe pneumonia, ARDS, sepsis, and septic shock.

The study was approved by the Turkish Ministry of Health and the Ankara City Hospital Ethical Committee.

### 2.1. Statistical analysis

In the statistical analysis of the data, SPSS 18.0 was used. Numbers and their percentages were used for categorical variables in descriptive statistics. Mean, standard deviation, median, and range (minimum–maximum) were used for numerical variables. For categorical variables, the chi-square test was used. When the chi-square condition was not met, Fisher’s exact test was used. Continuous variables were analyzed with Student’s t-test, and when parametric test conditions were not met, the Mann–Whitney U test was used. Logistics regression analysis was used for multivariate analysis. A P-value < 0.05 was considered statistically significant.

## 3. Results

A total of 222 (59.5% male, 40.5% female) patients were included in the study. Demographic and clinical characteristics of the patients are summarized in Table 1. Mean age was 50.6 ± 16.5 (18–93) years and mean age in the SARI/critical disease group was higher (47.7 ± 16.1 vs 62.2 ± 11.9 years, P < 0.001). Fifty (22%) patients had SARI or critical disease. Of the patients, 35% had a known contact with a COVID-19 patient. Of the patients, 12.6% were healthcare workers (HCW) employed in different hospitals in Ankara including Ankara City Hospital. Out of 26 HCW patients, 3 were in the SARI/critical disease group. All HCWs survived. Among all patients, 60 (27%) patients had history of travel abroad. Median time from illness onset to admission was 3 (0–21) days and was higher in SARI/critical disease group. At least one comorbidity was present in 92 (41.4%) patients. Presence of comorbidity, especially coronary artery disease and chronic obstructive pulmonary disease, was significantly higher in SARI/critical disease group (P < 0.05). Comorbidities are given in Table 2. Characteristics of the patients in terms of symptoms are given in Tables 1 and 3. Cough and fever were the most observed symptoms on admission, 58.1% and 48.6%, respectively. Anosmia and loss of taste were observed in 19 (8.6%) and 17 (7.7%) patients, respectively. Latest improved symptoms were fatigue, myalgia/arthralgia, loss of appetite, and dyspnea.

**Table 1:** Clinical and demographic characteristics of COVID-19 patients.

	All patients	Disease severity		
		Mild disease and pneumoniae (n = 172)	SARI and criticaldisease (n = 50)	P-value
Age (mean)	50.6 ± 16.5 (18–93)	47.7 ± 16.1	62.2 ± 11.9	<0.001
Age group				
<30 years	27 (12.2%)	26 (15.1%)	1 (2.0%)	<0.001
30-64 years	143 (64.4%)	120 (69.4%)	23 (46.0%)	
65-74 years	41 (18.5%)	20 (11.6%)	21 (42.0%)	
≥75 years	11 (5.0%)	6 (3.5%)	5 (10.0%)	
Age group				
>65 years	52 (23.4%)	26 (50.0%)	26 (50.0%)	<0.001
≤65 years	170 (76.6%)	146 (85.9%)	24 (14.1%)	
Sex				
Male (n = 137)	132 (59.5%)	99 (57.6%)	33 (66.0%)	0.285
Female (n = 92)	90 (40.5%)	73 (42.4%)	17 (34.0%)	
Days from illness onset to admission(median. min–max)	3 (0–21)	3 (0–15)	3 (0–21)	<0.001
Symptoms, no./total no (%) on admission				
Any symptom (at least one)	202 (91.0%)	153 (89.0%)	49 (98%)	0.052
Fever	108 (48.6%)	76 (44.2%)	32 (64.0%)	0.013
Cough	129 (58.1%)	96 (55.8%)	33(66%)	0.199
Sputum	4 (1.8%)	1 (0.6%)	3 (6.0%)	0.11
Sore throat	36 (16.2%)	34 (19.8%)	2 (4.0)	0.008
Runny nose	11 (5.0%)	11 (6.4%)	-	0.130
Dyspnea	60 (27.0%)	32 (18.6%)	28 (56.0%)	<0.001
Myalgia	37 (16.7%)	31 (16%)	6 (12.0%)	0.314
Nausea/vomiting	21 (9.5%)	15 (8.7%)	6 (12.0%)	0.582
Diarrhea	18 (8.1%)	16 (9.3%)	2 (4.0%)	0.376
Fatigue	55 (24.8%)	43 (25.0%)	12 (24.0%)	0.885
Loss of appetite	7 (3.2%)	6 (3.5%)	1 (2.0%)	1
Headache	31 (14.0%)	28 (16.3%)	3 (6.0%)	0.65

**Table 2:** Comorbidities of COVID-19 patients.

	All patients	Disease severity		
		Mild disease and pneumonia (n = 172)	SARI and criticaldisease (n = 50)	P-value
Any comorbidity	92 (41.4%)	63 (36.6%)	21 (42.0%)	0.007
Hypertension	52 (23.4%)	36 (20.9%)	16 (32.0%)	0.104
ACEI	19 (8.6%)	14 (8.1%)	5 (10.0%)	0.774
ARB	14 (6.3%)	8 (4.7%)	6 (12.0%)	0.091
ACEI or ARB	32 (14.4%)	22 (12.8%)	10 (20.0%)	0.201
Coronary artery disease	15 (6.8%)	8 (4.7%)	7 (14.0%)	0.048
Cardiovascular disease	56 (23.6)	36 (20.9%)	20 (40.0)	0.006
Diabetes mellitus	30 (13.5%)	20 (11.6%)	10 (20%)	0.127
COPD	12 (5.4%)	6 (3.5%)	6 (12.0%)	0.019
Obesity	77 (34.8%)	57 (33.0%)	20 (40.0%)	0.384
Chronic renal disease	6 (2.7%)	5 (2.9%)	1 (2.0%)	1
Malignancy	9 (4.1%)	6 (3.5%)	3 (6.0%)	0.425

ACEI: Angiotensin-converting-enzyme inhibitor, ARB: Angiotensin II receptor blocker, COPD: Chronic obstructive pulmonary disease

**Table 3:** Median Duration of symptoms in COVID-19 patients.

Symptom	Days
Fever	3 (1–20)
Cough	4 (1–20)
Dyspnea	4.5 (1–18)
Sputum	4.5 (2–10)
Sore throat	2 (1–11)
Runny nose	3 (2–11)
Myalgia/arthralgia	5 (1–14)
Nausea/vomiting	3 (1–12)
Diarrhea	3 (1–7)
Fatigue	5 (2–20)
Loss of appetite	5 (2–10)
Headache	3.5 (1–12)
Anosmia	4 (1–10)
Loss of taste	4 (1–7)
Chills	3(1–8)

Among 222 patients, 197 (88.7%) had positive PCR for SARS-CoV-2 on admission. The second samples taken 48 h after the first PCR test were positive in 25 (11.3%) patients. Median day for the first negative test after positive PCR was 5 (1–20). An influenza test was performed in 169 patients and only one (0.6%) patient had a positive result. On admission, 62 patients received oseltamivir treatment. Five (3.6%) patients had positive test results for another respiratory virus. Secondary bacterial infection was observed in 8 patients (3.6%). Overall mortality rate among these patients was 5.4%. Median age of mortal cases was 69 (44–81).

Complications are summarized in Table 4. Twenty-one complications occurred in 20 patients. Complications regarding thrombosis were observed in 4 patients. Immune thrombocytopenic purpura (ITP) occurred in one patient. Four patients who had complications were in the mild disease/pneumonia group.

**Table 4:** Complications of COVID-19 patients.

Any complication	21 (9.4%)
ARDS	12 (5.4%)
Pulmonary thromboembolic	3 (1.3%)
Septic shock	2 (0.9%)
Cerebral sinus venous thrombosis	1 (0.45%)
Rheumatic involvement	1 (0.45%)
Deep vein thrombosis	1 (0.45%)
Hepatitis (liver injury)	1 (0.45%)
ITP	1 (0.45%)

ARDS: Acute respiratory distress syndromeITP: Immune thrombocytopenic purpura

Laboratory findings of the patients are given in Table 5. Low lymphocyte count, high neutrophil/lymphocyte ratio, low monocyte count, low platelet count, elevated liver enzymes, low GFR, high levels of muscle enzymes, and high levels of ferritin and IL-6 on admission were found to be associated with SARI/critical disease (P < 0.05).

**Table 5:** Laboratory findings of COVID-19 patients on admission

	All patientsmedian (min–max)	Mild disease and pneumonia (n = 172)	SARI and critical disease (n = 50)	P-value
White blood cell count (×109/L)	5290 (1150–21850)	5170 (1150–19900)	5900 (3610–21850)	0.005
Neutrophil count (×109/L)	3300 (290–19800)	3180 (290–13400)	4300 (2300–19800)	<0.001
Lymphocyte count (×109/L)	1120 (150–4110)	1220 (380–4410)	770 (150–2090)	<0.001
Neutrophil/ Lymphocyte ratio	3 (0.4–38)	2.5 (0.4–28)	5.6 (1.5–38)	<0.001
Monocyte count (×109/L)	330 (100–1460)	330 (100–1460)	290 (100–1260)	0.036
Hemoglobin (g/L)		13.8 (7.1–17.3)	13.2 (8.6–16.7)	0.093
Platelet count (×109/L)	13.8 (7.1–17.3)	210.000(8.8000–499.000)	185.000 (100.000–406.000)	0.022
ALT, U/L	27 (7–408)	25 (7–123)	27 (9–408)	<0.001
AST, U/L	26 (7–321)	24 (7–122)	36 (14–321)	0.017
Total bilirubin (μmol/L)	0.5 (0.1–2.4)	0.5 (0.1–2.4)	0.6 (0.2–1.8)	0.024
Albumin (g/L)	41 (22–54)	44 (33–52)	40 (22–49)	<0.001
Creatinine (μmol/L)	0.85 (0.4–1.8)	0.82 (0.4–1.2)	0.95 (0.8–1.8)	<0.001
eGFR (mL/min/1.73m2)	93 (9–139)	97 (15–139)	77.5 (9–122)	<0.001
C-reactive protein (mg/L)	0.155 (0.005–0.252)	0.009 (0.005–0.240)	0.089 (0.03–0.252)	0.996
Procalcitonin (PCT) (μg/L)	0.05 (0.001–37)	0.03 (0.001–3.0)	0.46 (0.001–37)	0.411
Troponin (μg/L)	5.5 (1–56)	3 (2–56)	10 (1–42)	0.313
Myoglobin (μg/L)	53 (8–502)	33 (8–134)	141 (23–502)	0.042
T. cholesterol (mg/dL)	145 (45–211)	185 (159–211)	102 (90–114)	0.567
Triglyceride (mg/dL)	107 (10–319)	117 (111–123)	137 (80–94)	0.063
PT	12.4 (10.7–15.9)	12.2 (10.7–17)	13.0 (11.6–15.9)	0.552
aPTT	24.9 (19.3–33.5)	24.9 (20–33.5)	24.7 ± 2.9	0.859
INR	1.03 (0.9–1.37)	1.03 (0.9–2.6)	1.08 (0.98–1.37)	0.911
Ferritin (μg/L)	141 (0.96–1566)	96 (0.96–956)	433 (11–1566)	<0.001
D-dimer (μg/L)	2.1 (0.01–35)	0.33 (0.01–35)	0.95 (0.42–1.37)	0.141
Fibrinogen (g/L)	3.1 (1–8.6)	2.9 (1–7.3)	4.9 (2.3–8.6)	<0.001
Creatine Kinase (μ/L)	88 (16–5395)	82 (16–1161)	128 (29–5395)	0.016
LDH (U/L)	216 (122–892)	209 (122–622)	308 (153–892)	<0.001
IL-6 (pg/mL)	16.5 (2.4–168)	10 (2.4–49)	43 (4–168)	<0.001

Chest CT findings on admission of COVID-19 patients are given in Table 6. CT was normal in 26 (12.1%) patients. Consolidation was observed in 31 (14.4%) patients. Bilateral ground-glass opacity (52.1% vs 91.7%) and patchy infiltration (5.4% vs 22.9%) were more frequently seen in the SARI/critical disease group (P < 0.001). Unilateral ground-glass opacity was observed in 42 patients (25.1%) in the mild disease group, whereas it was not observed in any patient in the SARI/critical disease group.

**Table 6:** Chest CT findings on admission of COVID-19 patients.

	All patients	Disease severity		
	(n=215/222)7 patients had no CT	Mild disease and pneumonia (n=167/172)	SARI and critical disease (n= 48/50)	P-value
Normal	26 (12.1%)	142 (75.1%)	1/48 (3.8%)	0.012
Consolidation	31 (14.4%)	22 (13.2)	9 (18.8%)	0.332
Unilateral ground-glass opacity	42 (19.5%)	42 (25.1%)	0/48 (0%)	<0.001
Bilateral ground-glass opacity	131 (60.9%)	87 (52.1%)	44 (91.7%)	<0.001
Patchy infiltration	20 (9.3%)	9 (5.4%)	11 (22.9%)	<0.001
Crazy paving	21 (9.8%)	13 (7.8)	8 (16.7%)	0.066
Pleural effusion	4 (1.9%)	3 (1.8%)	1 (2.1%)	1
Nodular lesion	26 (12.1%)	21 (12.6%)	4 (8.3%)	0.41
Halo or reverse halo	13 (6.1%)	12 (7.2%)	1 (2.1%)	0.306
Air bronchogram	5 (2.3%)	5 (3.0)	-	0.589
LAP	17 (7.9%)	13 (7.8%)	4 (8.3)	0.901

LAP: lymphadenopathy

In the mild disease group, 5 (2.3%) patients received no treatment. Among all patients, 171 (77%) received hydroxychloroquine alone or in combination with azithromycin or doxycycline. Of the patients, 46 (23%) received favipiravir alone or in combination with hydroxychloroquine, azithromycin, or doxycycline. Drug-induced QT prolongation was observed in 3 (1.4%) patients who received hydroxychloroquine alone or in combination with azithromycin or doxycycline.

Supportive treatment regimens of the patients are given in Table 7. The most commonly used supportive treatment agent was tocilizumab (12 patients, 5.4%). Four patients receiving tocilizumab died. Oxygen therapy was required in 56 (25.2%) patients. Of the patients, 42 (18.9%) were followed up in the intensive care unit (ICU). Prone position and noninvasive mechanical ventilation were applied to 30 (13.5%) and 4 (1.8%) patients, respectively. Four patients among 30 patients followed with prone position died.

**Table 7:** Supportive treatment regimens of the patients.

	n (%)
Steroid	8 (3.6%)
Tocilizumab	12 (5.4%)
Anakinra	10 (4.5%)
Convalescent plasma	2 (0.9)
Mesenchimal stem cell	5 (2.3)
Intravenous Immunoglobulin	5 (0.3)
High dose vitamin C	11 (5.0)
Colchicine	11 (5.0)
Low molecular weight heparin (n=215)	89 (%41.3)

Independent risk factors for SARI/critical disease are given in Table 8. It was found that patients older than 65 years have 8-fold increased risk for development of SARI/critical disease.

**Table 8:** Independent risk factors for SARI and critical disease

	P value	Odds Ratio	95% C.I	
			Lower	Upper
Age (>65 years)	<0.001	8.071	3.362	19.374
Sex (male)	0.157	1.730	0.810	3.697
Hypertension	0.492	0.492	0.175	1.383
Coronary artery disease	0.621	1.439	0.340	6.092
COPD	0.134	2.822	0.728	10.944
Diabetes mellitus	0.524	1.398	0.499	3.913
Time for negative test after first symptom	0.787	0.989	0.914	1.070
Duration from illness onset to admission	0.519	0.964	0.862	1.078

Logistic regression analysis (“Enter” method. Nagelkerke R Sguare=0.226 ) COPD: Chronic obstructive pulmonary disease

## 4. Discussion

This study reveals the epidemiological and clinical characteristics, and the factors and parameters that determine the clinical course and prognosis of the COVID-19 patients admitted to the largest referral hospital in Europe. Since it is a referral hospital, this study contains valuable data in terms of the course of complicated patients and provides a wide range spectrum of clinical picture of COVID-19 patients.

Turkey has followed closely the developments related to the disease, and immediately implemented many preventive measures starting from the onset of the epidemic. These rapid and important actions have given time to be prepared for both the community and the health care system in Turkey during this pandemic. As of June 13, 2020, there are 176,677 cases and the mortality rate is 2.7% in Turkey. This mortality rate is lower when compared with the majority of countries across Europe, the Americas, and Asia. Since our hospital is a referral center for COVID-19 patients, the majority of the cases were transferred from secondary and tertiary care hospitals when their clinical conditions worsened. Consequently, the mortality rate reported in this study (5.4%) is higher than Turkey’s general mortality rate, but still lower than the global rate of 6.9%. Feng et al. reported a mortality rate in their study population that consists of COVID-19 patients from 3 different cities in China as 8% [2]. Richardson et al. reported 5700 hospitalized COVID-19 patients admitted to 12 hospitals in New York City [3] and found a mortality rate of 21%, and an ICU admission rate of 14.2%. In comparison, in this study, the mortality rate in Ankara City Hospital is considerably lower and ICU admission rate is higher (18.9%). Wang et al. reported that 26% of patients required intensive care unit treatment, and mortality was 4.3% [4]. These results might indicate that the transfer of patients to ICU at an appropriate time can be associated with low mortality. In many countries, mortality rates are higher than ICU admission rates. This may be a consequence of insufficient ICU beds. Turkey has the highest number of ICU beds per 100,000 citizens among highly populated countries. Ankara City Hospital was able to reserve 200 of its 800 ICU beds to COVID-19 patients and therefore was able to accept a high number of referral patients from healthcare facilities in Ankara and surrounding provinces. All patients deemed to require intensive care by their physicians were able to be transferred to an ICU in Ankara City Hospital without undue delay.

Severe disease in COVID-19 can occur in healthy individuals of any age. However, it is predominantly observed in adults with comorbidity like cardiovascular disease, diabetes mellitus, hypertension, chronic obstructive pulmonary disease, and malignancies [5]. In our study, at least one comorbidity was present in 92 (41.4%) patients. The presence of comorbidity, especially coronary artery disease and chronic obstructive pulmonary disease, was significantly higher in the SARI/critical disease group (P < 0.05). The CDC COVID-19 Response Team reported that among 7162 cases, 37.6% had one or more comorbidity or risk factors [6]. The presence of at least 1 underlying health condition or risk factor was found to be associated with a higher hospitalization and ICU admission rate. A metaanalysis reviewing 13 studies with 3027 patients revealed that hypertension, diabetes, cardiovascular disease, and respiratory disease were significantly higher in critical/mortal patients compared to noncritical patients, with odds ratios of 2.72, 3.68, 5.19, 5.15, respectively [7]. Since the angiotensin-converting enzyme 2 (ACE2) is the cellular receptor for SARS-CoV-2, discussions about the course of COVID-19 in patients using ACEI or ARB have been present since the beginning of the outbreak. In this study, we found no relationship between ACEI or ARB use and severity of COVID-19. In a large retrospective cohort study, prior ACEI/ARB use was not found to be associated with increased mortality [8]. In another similar study from Wuhan, authors concluded that taking ACEIs/ARBs is not a risk factor for severe disease or mortality [9]. In the current literature, there is insufficient evidence-based data revealing that ACEI/ARB use will worsen the course of COVID-19. Our results are in agreement with the current guidelines which recommend all patients who have started any ACE inhibitor/ARB to continue their medication.

In our patient population, age remains the only independent risk factor for SARI/critical disease. It was found that patients older than 65 years have an 8-fold increased risk for the development of SARI/critical disease. In a study comparing clinical characteristics of COVID-19 patients at different ages found that patients older than 60 years have heavier clinical course, poorer outcome, and longer disease courses compared with those under 60 years [10]. The CDC reported that the case-fatality rate increases with increasing age, from 0 among people aged up to 19 years to 10%–27% among people aged 85 years or more [6]. Xie et al. evaluated 168 fatal COVID-19 cases and reported that median age was 70 years, and 95.8% of these patients were older than 50 years [11].

There is no specific clinical feature that can reliably differentiate COVID-19 from other viral respiratory infections. The most common symptoms, fever and cough, have been reported at varying rates in different articles. In our study, cough and fever were the most observed symptoms on admission, 58.1% and 48.6%, respectively. The duration of symptoms is an important parameter for clinicians. In our cohort, the most rapidly resolving symptom was sore throat with median 2 days (1–11 days), and the frequency of sore throat was significantly higher in the mild disease group when compared to SARI/critical disease group (19.8% vs. 4%). Although the first reported cases had isolated respiratory symptoms, with the progression of the pandemic and the increasing number of patients it was observed that olfactory and gustatory dysfunction, which indicates nervous system involvement, were also important symptoms of COVID-19. In a systematic review and metaanalysis regarding olfactory and gustatory dysfunction in COVID-19 patients, the prevalence of anosmia and taste disorder were found to be 52.73% (95% CI, 29.64%–75.23%) and 43.93% (95% CI, 20.46%–68.95%), respectively [12]. In our study, anosmia and loss of taste were observed 8.6% and 7.7% of the patients, respectively. These symptoms may have been overlooked since it is unlikely for someone who is seriously ill to notice the loss of taste and smell unless specifically asked. Although there is no specific symptom in COVID-19, the coexistence of some symptoms like fever and anosmia or loss of taste can increase the likelihood of diagnosis.

Determining the laboratory tests that will contribute to the follow-up of COVID-19 patients has an important role for identifying severe and nonsevere cases or patients with low or high mortality risk. Leukocytosis, an increase in neutrophils and a decrease in lymphocytes, monocytes, and eosinophils can be observed in COVID-19 [13,14]. These findings can be monitored by an increase in the neutrophil/lymphocyte ratio (NLR). We found that median NLR on admission are 2.5 (0.4–28) in the mild disease/pneumonia group and 5.6 (1.5–38) in the SARI/critical disease group. This ratio can help physicians to predict the patient’s prognosis. In a retrospective, multicenter cohort study, lymphocyte count was significantly higher in nonfatal cases when compared to fatal cases; in survivors, lymphocyte count improved after 7th day of illness whereas severe lymphopenia continued until death in patients who died [15]. We found that elevated liver enzymes, low GFR, high levels of myoglobulin and creatine kinase, and high levels of ferritin and IL-6 on admission were found to be associated with SARI/critical disease (P < 0.05). Zhang et al. reported that IL-6 levels were 5 pg/mL and 35 pg/mL in discharged and deteriorated patients, respectively [16]. In our patients, median and highest levels IL-6 levels on admission were 10 pg/mL and 49 pg/mL in mild disease/pneumonia group and 43 pg/mL and 168 pg/mL in SARI/critical disease group. These results indicate that high levels of IL-6 can be an early warning for cytokine storm and poor prognosis on admission. All severe COVID-19 cases should be monitored for hyperinflammation. It is important to recognize a life-threatening cytokine storm early and identify subgroups that may benefit from immunosuppressive agents. The most used supportive treatment agent was tocilizumab (12 patients, 5.4%) in our study.

The most unfavorable prognostic symptom in COVID-19 is the development of coagulopathy and its incidence has not been established. Like in sepsis, complement activation, endothelial damage, and inflammatory and microthrombotic pathway activation can predispose to thrombosis. In our study, complications regarding thrombosis were observed in 4 patients. Wichmann et al. found deep venous thrombosis in 7 of 12 patients (58%) during autopsies of COVID-19 cases where no venous thromboembolism was suspected before death; the cause of death was determined as pulmonary embolism in 4 patients [17].

Supportive treatment remains vital for COVID-19 patients. Clinicians should monitor patients closely for the need for oxygen therapy and intensive care. Oxygen therapy was required in 56 (25.2%) of our patients and among 222 patients, 42 (18.9%) were followed up in the ICU.

In conclusion, this first cohort study regarding COVID-19 cases in Turkey reveals the epidemiological and clinical characteristics, and the factors and parameters that determine the clinical course and prognosis of the COVID-19 patients admitted to the largest referral hospital in Europe. Older age (>65), presence of comorbidity (especially coronary artery disease and chronic obstructive pulmonary disease), bilateral ground-glass opacity and patchy infiltration on CT, low lymphocyte count, high neutrophil/lymphocyte ratio, low monocyte count, low platelet count, elevated liver enzymes, low GFR, high levels of muscle enzymes, and high levels of ferritin and IL-6 on admission are predictors of SARI and severe disease.
